# Estimating the cost of COVID-19 vaccine deployment and introduction in Ghana using the CVIC tool

**DOI:** 10.1016/j.vaccine.2022.01.036

**Published:** 2022-03-15

**Authors:** Justice Nonvignon, Richmond Owusu, Brian Asare, Alex Adjagba, Yap Wei Aun, Karene Hoi Ting Yeung, Joycelyn Naa Korkoi Azeez, Martha Gyansa-Lutterodt, Godwin Gulbi, Kwame Amponsa-Achiano, Frederick Dadzie, George E. Armah, Logan Brenzel, Raymond Hutubessy, Stephen C. Resch, Ghana Health Technology Assessment Technical Working Group

**Affiliations:** a Department of Health Policy, Planning and Management, School of Public Health, University of Ghana, Legon, Ghana; b Ministry of Health, PMB, Accra, Ghana; c Health Section, UNICEF, 3 UN Plaza, New York, NY 10017, USA; d Quanticlear Solutions, Malaysia; e Department of Immunization, Vaccines and Biologicals, World Health Organization, Geneva, Switzerland; f Expanded Programme on Immunization, Ghana Health Service, Accra, Ghana; g Noguchi Memorial Institute for Medical Research, University of Ghana, Accra, Ghana; h Bill & Melinda Gates Foundation, Seattle, WA, USA; i Center for Health Decision Science, Harvard T.H. Chan School of Public Health, 718 Huntington Ave., Boston, MA 02115, USA

**Keywords:** COVID-19 vaccine, Costs, CVIC tool, Ghana

## Abstract

**Background:**

This study estimated cost of COVID-19 vaccine introduction and deployment in Ghana.

**Methods:**

Using the WHO-UNICEF COVID-19 Vaccine Introduction and deployment Costing (CVIC) tool Ghana’s Ministry of Health Technical Working Group for Health Technology Assessment (TWG-HTA) in collaboration with School of Public Health, University of Ghana, organized an initial two-day workshop that brought together partners to deliberate and agree on input parameters to populate the CVIC tool. A further 2–3 days validation with the Expanded Program of Immunization (EPI) and other partners to finalize the analysis was done. Three scenarios, with different combinations of vaccine products and delivery modalities, as well as time period were analyzed. The scenarios included AstraZeneca (40%), Johnson & Johnson (J&J) (30%), Moderna, Pfizer, and Sputnik V at 10% each; with primary schedule completed by second half of 2021 (Scenario 1); AstraZeneca (30%), J&J (40%), Moderna, Pfizer, and Sputnik V at 10% each with primary schedule completed by first half of 2022 (Scenario 2); and equal distribution (20%) among AstraZeneca, J&J, Moderna, Pfizer, and Sputnik V with primary schedule completed by second half of 2022 (Scenario 3).

**Results:**

The estimated total cost of COVID-19 vaccination ranges between $348.7 and $436.1 million for the target population of 17.5 million. These translate into per person completed primary schedule cost of $20.9–$26.2 and per dose (including vaccine cost) of $10.5–$13.1. Again, per person completed primary schedule excluding vaccine cost was $4.5 and $4.6, thus per dose excluding vaccine also ranged from $2.2 – $2.3. The main cost driver was vaccine doses, including shipping, which accounts for between 78% and 83% of total cost. Further, an estimated 8,437–10,247 vaccinators (non-FTEs) would be required during 2021–2022 to vaccinate using a mix of delivery strategies, accounting for 8–10% of total cost.

**Conclusion:**

These findings provide the estimates to inform resource mobilization efforts by government and other partners.

## Highlights

• Current COVID-19 vaccine supply market means LMICs will have to rely on a combination of different sources/types of vaccines to meet their demand.
• Deployment of COVID-19 vaccine plans in Ghana will cost $348.7–$436.1 million for coverage of 17.5 million of eligible Ghanaians.
• Vaccine cost constitute 78–83% of total cost whereas the total vaccination cost is 0.48–0.60% of the country’s 2020 GDP.
• The WHO-UNICEF CVIC tool is useful for comprehensive COVID-19 vaccine deployment costing and resource planning.

## 1 Introduction

Countries across the world have been battling to contain the spread of the COVID-19 pandemic since early 2020. As of 21st January 2022, about 340.5 million confirmed cases of COVID-19 were reported with more than 5.5 million deaths globally [1]. In Ghana, the first two cases of COVID-19 were recorded on the 12th of March 2020, with 155,665 cases and 1,370 deaths recorded as at 18th January 2022 [2]. In low and middle-income countries (LMICs), the economic and health impacts of the pandemic have been enormous. For instance, the World Bank estimated that the pandemic led to an average of 2% reduction in gross domestic product (GDP) in sub-Saharan Africa (SSA) in 2020 [3]. COVID-19 has plunged the region into its first recession in over 25 years, pushing about 40 million people into extreme poverty; a situation that has erased at least five years of progress in fighting poverty [3]. Worse yet, per capita output is not expected to return to 2019 levels until after 2022—in many countries, per capita incomes will not return to precrisis levels before 2025 [3]. The already weak health systems of LMICs, particularly SSA, worsened the situation in many countries. In Ghana, 2020 GDP was 1.7%, falling from the 6.5% reported in 2019, the year preceding the pandemic [4]. Further, a 21-day stay-at-home order in selected cities is estimated to have likely caused about 28% decrease in GDP during that period; and made about 3.8 million Ghanaians temporarily become poor [5].

In the health system, costs including medical costs, post-infection care, etc. are huge, and when these are coupled with productivity losses due to absenteeism and premature deaths the economy suffers. The economy of Ghana has suffered enormously, despite the health, economic and social measures that the government introduced to improve the situation impacted by the pandemic on households and businesses [6]. In order to prevent the spread of COVID-19 different public health and social measures are adopted including wearing of face masks, social and physical distancing, stay-at-home order, etc. To prevent domestic and international spread, global effort was channeled to development of vaccines to control spread and reduce morbidity and mortality. For many infectious diseases, vaccines are the best tools available for control and elimination [7]. Every year, vaccines avert four to five million deaths [8], and they are often touted as one of the most cost-effective health interventions [9], [10], [11], [12], [13], [14]. At the global level, a total of over 9.5 billion vaccine doses had been administered as at 19th January 2022 [1]. In the African region, coverage is reported to be as low as 15.5% for people who have received at least one dose and 10.4% for those who have completed primary schedule [56]. Following global efforts, the government of Ghana introduced COVID-19 vaccination in March 2021 in line with global strategy to control COVID-19. As of 18th January 2022, a total of 9.3 million of vaccine doses have been administered on an eligible total population of 17.5 million with 15.7% of the people completing primary schedule in Ghana [2].

Vaccination as a public health system intervention against diseases have huge economic and cost implications for countries. Similarly, the roll out of the unprecedented COVID-19 vaccine introduction and deployment to large populations will present financial burden to already stressed health systems in LMICs. Against this backdrop, it is necessary for countries to realistically plan and anticipate the resources required for successful roll out and sustainability of COVID-19 vaccination for their populations and in order to budget and finance the COVID-19 National Deployment and Vaccination Plan (NDVP) appropriately. In effect, Ghana used the COVID-19 Vaccine Introduction and deployment Costing (CVIC) tool as part of its planning and implementation of COVID-19 roll out, including funds raising efforts at the international level. In this paper, we present estimates of the cost of COVID-19 vaccine deployment and introduction in Ghana in 2021 to 2022, with a view to informing Ghana’s response and vaccine planning efforts, as well as guiding other countries. This is the first example of application of the CVIC tool being summarized and published.

## 2 Material and methods

### 2.1 The CVIC tool

The WHO and UNICEF introduced the CVIC tool that aims at guiding countries that seek to introduce COVID-19 vaccines.^2^ The tool provides a platform by which costs related to COVID-19 vaccination may be comprehensively costed. It is used to estimate the incremental costs for domestic and external resource mobilization purposes, including the World Bank’s COVID-19 Fast-Track Loan Facility. Countries can also use the tool to prepare budgets for vaccination beyond 2021 as COVID-19 vaccine is deployed. The tool aligns with the COVID-19 vaccine introduction readiness assessment tool^3^ (VIRAT) and the guidance on developing a NDVP,^4^ as well as the WHO SAGE values framework for the allocation and prioritization of COVID-19 vaccination [15].

The CVIC tool is Microsoft Excel-based, with as much as possible data pre-populated – using available country-specific data from global databases (e.g., Global health observatory, UN world population, etc.). The tool is, by default, set up to support a rapid cost estimate in a modular manner. Table 1 lists the subsections of the four mandatory input sections in the CVIC tool.

**Table 1:** Four mandatory input sections in the CVIC tool.

Input sections	Subsections
Population and delivery	Total population; essential workers; sociodemographic/health status and groups at risks distribution; delivery modalities; human resources for health (HRH); vaccine supply; selection and expected uptake
Unit costs	HRH; vaccine-related supplies, freight, cold storage and cold chain; local data management, monitoring, and pharmacovigilance; local demand generation and communications; and security
Central costs	Planning and coordination; budgeting; regulatory; prioritization, targeting, and COVID-19 surveillance; service delivery; training and supervision; monitoring and evaluation; vaccine procurement, cold chain, logistics, and infrastructure; vaccine safety surveillance; demand generation and communications
Target population	Distribution of uptakes in each half of the year of vaccination

In this exercise, version 2.1 of the CVIC tool^5^ was used. Over an eight-month period spanning February – September, 2021, data gathering, data analysis, and stakeholder engagement were undertaken. Foremost, to estimate COVID-19 vaccination costs, the Ministry of Health’s Technical Working Group for Health Technology Assessment (HTA) organized a two-day workshop, on 22nd and 23rd February 2021 at Alisa Hotel, that brought together relevant stakeholders. These included people from National Immunization Technical Advisory Group (NITAG), Ministry of Health (MOH), Ghana Health Service (GHS), UNICEF, WHO, Expanded Program of Immunization (EPI), National Health Insurance Authority (NHIA), University of Ghana School of Public Health, etc. Individuals from these bodies were purposively selected because they were in a position to provide relevant data to populate the tool. It is also worth emphasizing that, relevant information was retrieved from Ghana’s National COVID-19 vaccine Deployment and Vaccination Plan (NCvDVP). Over the two-day period, the mandatory sections of the tool – *population and delivery, unit costs, central costs,* and *target populatio*n were adequately completed.

On 23rd June 2021 at the MOH office, preliminary results from scenarios analysis were presented to the special session of the Ghana HTA Steering Committee, organized by the HTA Secretariat. In attendance were the WHO country Representative, the Lead of the Presidential Taskforce on COVID-19, and the Presidential Advisor on Health, alongside other officers from the MOH, Ghana Health Service, and partners. The meeting was used to discuss key input parameters that needed re-examining. The final stage of the exercise was the two-day meeting on 8th and 9th July 2021 at the EPI head office, with the EPI team together with its partners (WHO, UNICEF, JSI, PATH, etc.) where additional logistical needs were updated including ultra-cold chain equipment upgrade to account for the inclusion of Pfizer in the revised COVID-19 vaccine NDVP. Subsequently, the data was analyzed in the tool and produced relevant output that is discussed below. It must be noted that all financial and economic costs are reported in 2021 United States Dollars (US$) where US$1.00 = GH₵5.76; i.e., the exchange rate in the CVIC tool (version 2.1).

### 2.2 Target population for vaccination

The target populations (i.e., all Ghanaians aged 16 years and above and are not pregnant are eligible for vaccination and they will be reached through different delivery modes outlined below using two doses for all vaccines (AstraZeneca, Pfizer, Moderna, Sputnik V) and one dose for Johnson & Johnson (J & J) vaccine. The country’s NDVP clearly prioritizes key populations including the elderly (60 + years), healthcare workers, and essential workers (i.e., security personnel, utility service workers, etc.). This is consistent with WHO SAGE roadmap for prioritizing use of COVID-19 vaccines [15].

### 2.3 Delivery modalities

In the estimation of the cost of deployment, a combination of immunization service delivery modalities available in Ghana were considered. These include fixed sites with cold-storage (Modality 1), campaigns at fixed sites with no cold-storage (Modality 2), residential institutions (Modality 3), and remote Outreach & Mobile sites requiring overnight, multiday outreach (Modality 4). The separation of these delivery modalities was necessary because each of them has different cost implications. It is noteworthy that delivery modality 3 (e.g., nursing homes for older persons) is unavailable in Ghana, and therefore was excluded from the analysis. Health care workers are responsible for vaccination with support from volunteers who are integral part of the vaccination exercise in Ghana as they contribute to social mobilization activities.

### 2.4 Scenarios

Table 2 below presents the various assumptions underlying the scenarios considered. Three main scenarios were analysed. These were based on two overarching assumptions; the types of vaccines to be used and the time period over which the target population is expected to complete primary schedule. It must be noted that, some of the assumptions follow Ghana’s COVID-19 vaccine NDVP, for example, the assumption of vaccinating the entire eligible population (17.5 million) by 2021 [16]. However, given the global situation surrounding the vaccine market, full coverage of vaccination was more likely to happen in 2022. The determination of the price of vaccines for the analysis was obtained from UNICEF’s COVID-19 Vaccine Market Dashboard.^6^ Given that different countries are buying the vaccines at different prices, the team decided to use the median prices for the various vaccines. The main vaccines considered in this analysis are AstraZeneca, Sputnik V, Moderna, Pfizer, and J & J.

**Table 2:** Assumptions underlying the various scenarios.

Scenario		Vaccine type (%)				2021 (%)		2022 (%)	
	AstraZeneca	Sputnik V	Moderna	Pfizer	J & J	H1	H2	H1	H2
1	40	10	10	10	30	2.7	97.3	–	–
2	30	10	10	10	40	2.7	67.3	30	–
3	20	20	20	20	20	2.7	47.3	30	20
Median Prices	$4	$19	$18	$14	$10				

*H1 and H2 – first half and second half of the year respectively.

In Scenario one, the majority of vaccines was assumed to be AstraZeneca (40%), followed by J & J (30%), and the remaining 30% was assumed to be equally distributed between Moderna, Pfizer, and Sputnik V at 10% each. In Scenario two, it was assumed to have 30% AstraZeneca, 40% J & J, and 10% each for Moderna, Pfizer, and Sputnik V. In Scenario three, the assumption was equal distribution (20%) among AstraZeneca, J & J, Moderna, Pfizer, and Sputnik V. The vaccines considered in the scenarios were those that had been approved by Ghana’s Food and Drugs Authority (FDA). It is worth emphasizing that by the time of this analysis (July 2021), Ghana had covered only 2.7% of the target population in the first half of 2021.

Table 3 presents the key input parameters used in the estimation, along with the estimates.

**Table 3:** Key Input Parameters.

Parameter	Price ($)/Quantity	Source
Low Dead Space Syringe (0.3 ml) AD	0.05	CVIC tool
Syringe (0.5 ml) AD	0.05	CVIC tool
23G needle	0.01	CVIC tool
Alcohol Swab, Plaster, Dry Swabs	0.01	CVIC tool
21G needle	0.01	CVIC tool
Sodium Chloride 0.9% ampule 10mls	0.05	CVIC tool
RUP syringe (3 ml)	0.06	CVIC tool
Sharps Disposal (5L)	0.84	CVIC tool
Vaccination Card	0.03	CVIC tool
Airfreight price - First 100 kg	10	CVIC tool
Airfreight price - Above 100 kg	6	CVIC tool
Ground handling costs per shipment	200	CVIC tool
Electronic temperature monitoring device (leave blank for default cost)	40	CVIC tool
Wastage of vaccine	5%	CVIC tool
COVAX Facility contribution (Vaccine) (% of 2020 total population)	20%	CVIC tool
Population parameters	Quantity	
Total population	31,732,126 *^(2021)^* 32,395,456 *^(2022)^*	UNWPP 2019
Target population to be vaccinated	17,459,408	Ghana’s NCvDVP, 2021
Essential workers (e.g., security, utility service providers)	13.9%	Ghana’s NCvDVP, 2021
Healthcare workers	0.65%	Ghana’s NCvDVP, 2021
Groups with comorbidities or health states determined to be at significantly higher risk of severe disease or death	4%	Ghana’s NCvDVP, 2021

### 2.5 Sensitivity analyses

A multi-way sensitivity analysis was performed by varying key parameters including vaccine combinations (quantities) and time lines for primary schedule completed of target population. In addition, the CVIC tool provides upper and lower uncertainty bounds for cost estimates based on variation in the time taken by a vaccinator to vaccinate a person. The three scenarios were considered to be part of the sensitivity analysis.

## 3 Results

### 3.1 Target population

Ghana planned to vaccinate the entire eligible population in the country by the end of 2021. However, given the global situation surrounding the vaccine market, the achievement of this goal delayed to 2022. To that end, an estimated 17.5 million of the population are considered to be eligible and would be vaccinated during this period (2021–2022). The projections show that cumulatively 16.7 million (52%) of the total population (32.4 million; 2022 est.) will be vaccinated by end of 2022 given the expected uptake rates. Even though, the target is 17.5 million, some people may not volunteer to vaccinate.

### 3.2 Total cost

Table 4 below shows that the total cost of COVID-19 vaccine deployment and introduction is estimated to range from $348.7 million (Scenario 1) to $436.1 million (Scenario 3). These translate into per person completed primary schedule cost of $20.9 to $26.2. Again, per person completed primary schedule excluding vaccine cost between $4.5 and $4.6, thus, per dose excluding vaccine also ranged from $2.2 to $2.3 (Table 4).

**Table 4:** Total Cost of COVID-19 Vaccine Introduction and Deployment in Ghana in 2021–2022.

Scenario	Description				2021		2022		Total ($ mil)
	Per person completed primary schedule ($)	Per person completed primary schedule excluding vaccine ($)	Per dose including vaccine ($)	Per dose excluding vaccine ($)	H1 ($ mil)	H2 ($ mil)	H1 ($ mil)	H2 ($ mil)	
1	*20.9*	*4.5*	*10.5*	*2.2*	33.1	315.6	–	–	**348.7**
2	*21.2*	*4.6*	10.6	2.3	31.4	209.0	112.9	–	**353.3**
3	*26.2*	*4.6*	*13.1*	*2.3*	31.4	238.4	87.0	79.4	**436.1**

*H1 and H2 – first half and second half of the year respectively.

### 3.3 Cost breakdown by category

**Fig. 1:**
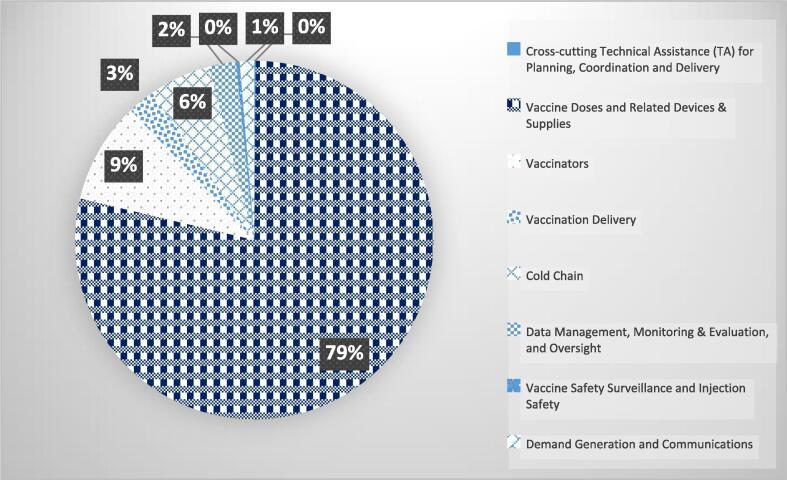
Cost breakdown – Scenario 1.

**Fig. 2:**
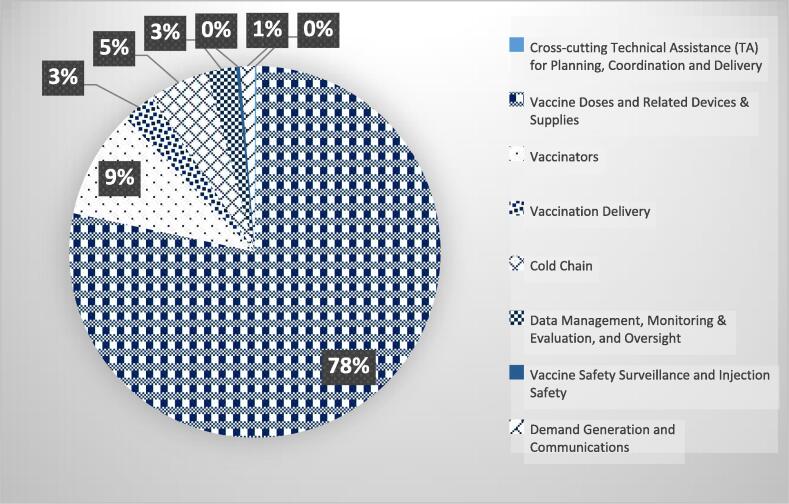
Cost breakdown – Scenario 2.

**Fig. 3:**
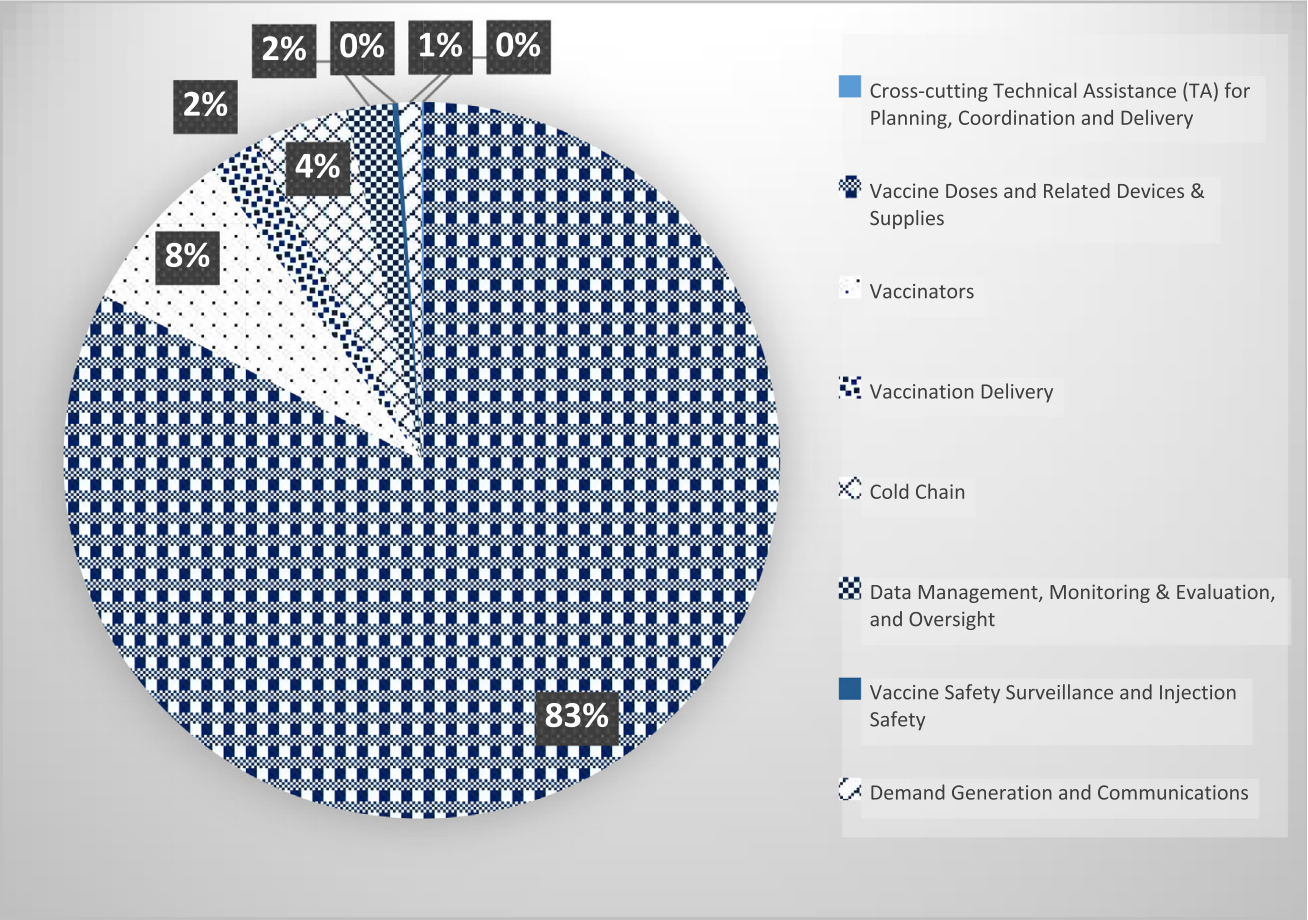
Cost breakdown – Scenario 3.

**Fig. 4:**
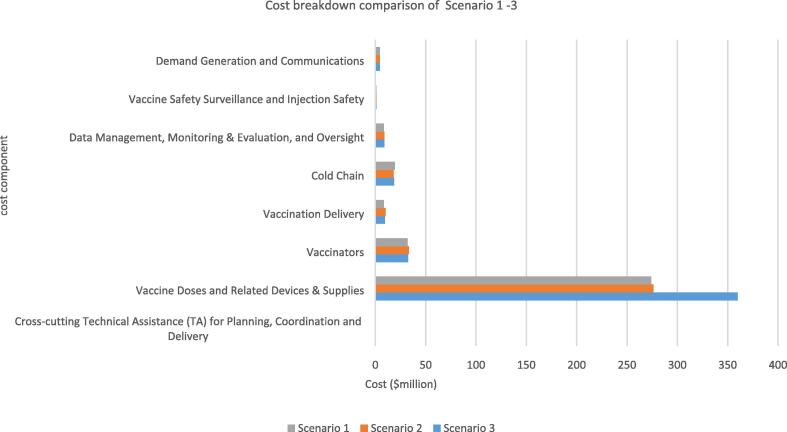
Cost breakdown comparison of Scenario 1–3.

As shown in Fig. 1, Fig. 2, Fig. 3, the main cost driver is vaccine procurement cost, including shipping, which accounts for between 78% (scenario 2) to 83% (scenario 3) of total cost. This is followed by the human resources for health including vaccinator training, deployment, and compensation, which ranged between 8% and 10% of total cost. Other minor contributors to cost are cold chain (4–6%) and data management and monitoring, pharmacovigilance, and oversight (2–3%). On the other hand, the least cost drivers were domestic logistics and transport, excluding cold chain, security, and stand-alone technical assistance (Fig. 1, Fig. 2, Fig. 3). In Fig. 4, the cost breakdown of the three scenarios is compared.

Fig. 4 shows a comparison of the cost breakdown for the three scenarios.

### 3.4 Human resource and relative HRH impact

Human resources for health are crucial for immunization activities. Regarding the three delivery modalities that Ghana adopts, majority of the personnel (not FTEs) was estimated to be for outreach (n = 4,128–4,768). For fixed sites with cold storage, the required number of vaccinators (non-FTE) was estimated to be the same for all three scenarios (n = 2,554). However, for campaigns at fixed sites with no cold-storage the numbers ranged from 1,755 – 2,925 (Table 5). In all, a maximum of between 8,437 (Scenario 3) and 10,247 (Scenario 1) of vaccinators (non-FTEs) would be required (Table 5). The cadre of health care workers used in Ghana for this exercise were nurses (i.e. general nurses, community health nurse, public health nurses), and disease control officers. Data capture and entry would be undertaken by health information officers.

**Table 5:** Human Resources Requirements for various delivery modalities and Overall Impact of the Vaccination on Health Workforce in Ghana.

**Item**	**Scenario 1**	**Scenario 2**	**Scenario 3**
Delivery Modality 1	2,554	2,554	2,554
Delivery Modality 2	2,925	1,833	1,755
Delivery Modality 4	4,768	4,128	4,128
***Total Current vaccinators (non-FTEs)***	**10,247**	**8,515**	**8,437**
Delivery Modality 1	1,415	943	904
Delivery Modality 2	2,858	1,791	1,715
Delivery Modality 4	2,751	2,382	2,382
***Total Full-time Equivalents***	**7,023**	**5,115**	**5,000**
***Total Vaccinator days***	**938,038**	**979,265**	**959,532**
HRH supply impact (non-FTEs)	19.7%	16.4%	16.2%
HRH supply impact (FTEs)	13.5%	9.8%	9.6%

In addition, a maximum of 5,000 – 7,023 vaccinators (FTEs) would be required, with many being in outreach delivery (Table 5). Elsewhere, it was estimated that 938,038 – 979,265 vaccinator days would be used during the period under review (2021 – 2022); this is largely dominated by vaccination using the outreach modality (Table 5). The rollout of this COVID-19 vaccination would impact human resource for health supply. Table 5 shows that Current Vaccinators (non-FTEs) would impact 16.2% − 19.7% of HRH supply and current vaccinators (FTEs) would impact HRH supply by 9.6% − 13.5%.

## 4 Discussion

### 4.1 Cost of COVID-19 vaccine introduction and deployment

Ghana targeted vaccinating 17.5 million of the population against the COVID-19. This translates into about 54% of the total estimated population for 2021. However, about 16.7 million (52%) of the total population may be successfully vaccinated over the projected vaccination period (2021 – 2022). The success of COVID-19 vaccination programs in combination with public health and social measures depends on governments’ goal. In the case of Ghana, both health and economic goals are the drive for the COVID-19 vaccination roll out. Health goals include prevention of COVID-19 related infections, deaths, and negative health system impact while economically, the country aimed “to protect livelihood, restore economic activities and put the country on course to recover and build back better as indicated in Ghana’s NCvDVP” [16].  

The country’s NCvDVP clearly prioritizes key populations including the elderly (60+ years), healthcare workers, and essential workers (i.e., security personnel, utility service workers, etc.). The prioritization of healthcare workers is to protect the healthcare system from collapse, while for the elderly, they have emerged as the most vulnerable in terms of disease severity and deaths [17], [18], [19], [20]. Overall, the country’s prioritization strategy is congruent with the WHO SAGE roadmap for prioritizing use of COVID-19 vaccines [21]. It is noteworthy that earlier COVID-19 vaccination excluded children, a phenomenon which has changed in many countries over the last few months. In the US and Europe, for example, emergency approval for use of COVID-19 vaccine in children was granted in the last quarter of 2021 [22], [23], [24]. This means that countries would have to revise their vaccination target population to include children. In Ghana, inclusion of children in the COVID-19 vaccination has huge cost implications for government because a significant proportion (greater than 40%) of the population represents children < 16 years who were originally excluded from the current COVID-19 NDVP being implemented [16]. In effect, the government of Ghana would have to raise additional funds to cater for the cost vaccinating children in order to achieve the overall goal of COVID-19 vaccination rollout in Ghana. Moreover, the development of new variants and the consequent adoption of booster vaccinations presents additional cost which may have to be considered by government in the future as part of comprehensive effort to control COVID-19 in Ghana.

The total cost of vaccination was estimated to be between $348.7 – $436.1 million depending on the combination of vaccine products and timelines the government chose. The estimated total cost of vaccination was roughly equivalent to 60.9–76.1% of the total non-remuneration budget allocation to the health sector ($572.9 million) [25]. Globally, the economic impact of COVID-19 has been overwhelming, with developing countries suffering further on their already weak economic and fiscal foundations [26], [27], [28], [29], [30]. Yet, it is imperative that governments make the effort to vaccinate the population in order to fully reopen their economies. The income level of a country may be the best guide for such decision. Ghana currently has a GDP per capita of $2,328.5 [31]. Moreover, the total cost of vaccination is 0.48–0.60% of Ghana’s 2020 GDP [32]. This is relatively favorable for a $20.9–$26.2 cost of person completed primary schedule. This is about 26.8–33.6% of the 2018 per capita health expenditure in Ghana [33]. Again, in addition to the COVAX facility which guarantees support for up to 20% of the population [34], and other support and pledges from bilateral and multilateral partners, support could be solicited from other domestic and external sources. It is important to emphasize that it will take full political will and commitment to finance this vaccination programme by government. Thus, against the backdrop of a full appreciation that the short- and long-term health and economic benefits of this programme far exceed the immediate financial outlay by government.

Elsewhere, using an assumption of the price of COVID-19 vaccine per dose at least equal to the average price of a seasonal flu vaccine ($36), Meskarpour-Amiri et al. [35] estimated the cost of COVID-19 vaccination. The authors report that financing at least one dose of vaccination in Ethiopia, Guinea, Niger, and Chad will exceed these countries’ total health expenditure in one year. Similarly, countries such as Afghanistan, Pakistan, India, and Ghana have to spend more than half of their total health expenditure on one dose of vaccine, which is unaffordable [35]. Specifically for Ghana, Meskarpour-Amiri et al. [35] estimated that $2.19 billion will be needed to cover the entire population for a two dose COVID-19 vaccination and accounting for the targeted 52% of the total population that our analysis produced, Meskarpour-Amiri et al. [35] estimate translates into about $1.26 billion, which exceeds the general government health expenditure ($1.04 billion) [36]. It is noteworthy, that these estimates are more than three times what this study found (Table 4). Interestingly, their price assumption for vaccine per dose are significantly higher than the current prevailing market prices [37]. Moreover, they did not use the CVIC tool which is viewed as more comprehensive and needs country-specific data to produce the necessary context-specific results.

The results of this study indicate that vaccine doses and related devices and supplies account for 78–83% of total vaccination costs. Foremost, there are some differences between the three scenarios in this study, mainly driven by the variation in the prices and number of doses. For example, the lowest price is the $4.00 per dose for AstraZeneca while the highest price per dose is $19.00 for Sputnik V. Because Sputnik V is a two-dose vaccine, the total cost for the vaccine alone is $38.00 compared with $8.00 for a two-dose AstraZeneca vaccine. In several other vaccine introduction in other countries – Egypt, Paraguay, Iran, Bangladesh, and Ghana, the cost of vaccine accounts for a large part of total cost of vaccination programmes [38], [39], [40], [41], [42]. The expenditure on vaccinators is estimated to be 8–10% of total costs; making this item the next most significant cost driver for the vaccination programme. This cost component is in two major forms – the direct financial compensation for vaccinators and the cost of their training activities. Clearly, beyond the vaccine availability, this is one of the main determinants of the success of every vaccination programme. In this cost analysis, cold chain emerged an important cost driver contributing to between 4% and 6% of total cost. This is mainly because of the development that additional cold chain equipment for ultra-cold storage (e.g., walk-in cold rooms, walk-in refrigerators/freezers, etc.) are required for vaccines such as Pfizer and Moderna. These additional cold chain infrastructural requirements that need to be procured may have accounted for this.

### 4.2 Financing of COVID-19 vaccine

With the level of global attention given to COVID-19 vaccine procurement and implementation, the distribution and financing of COVID-19 vaccines, particularly in LMICs, have become topical issues [43], [44], [45], [46], [47], [48], [49], [50], [51]. This discourse mainly is on the backdrop of concerns over equity in access to vaccines in LMICs, following from similar issues relating to H1N1 influenza in 2009 [52]. The arsenal of funding sources for COVID-19 vaccines varies depending on the country, its geographic location, and the economic ensemble that it belongs, Gavi eligibility, World Bank status for grants, etc. UNICEF estimates potential financing gap of between $1.3 – 6.9 billion in 133 countries based on different scenarios [53]. In Africa, the Africa Centre of Disease Control and Prevention (CDC-Africa) estimates that Africa needs about 1.5 billion doses of the vaccines in order to vaccinate 60% of the population to contain the pandemic, that is, to arrest further transmission and death from COVID-19 [54]. This will cost approximately $10–15 billion [54] which is expected to come from various sources including governments, bilateral and multilateral partners such as the World Bank, and African Import Export Bank (Afrexim), and the COVAX Donor Initiative, and other stakehoders. Also, measures such as the Access to COVID-19 Tools (ACT) Accelerator to promote equitable access to vaccines, therapeutics, and diagnostics have been put in place by the WHO and partners [55]. The COVAX Facility only represents 20% of total vaccine needs of participating countries [57], which means that countries have the responsibility of financing the remaining vaccine gap. For the COVAX Facility that seeks to provide two billion doses of vaccines for participating countries in 2021, there are concerns about its attainability because of vaccine nationalism by high income countries [48].

Despite the current arrangement of support from COVAX Facility, the financing of the 80% of the population and the need to improve infrastructure as it is the case of Ghana means COVID-19 vaccine financing will continue to put financial pressure on LMICs’ budget. Countries like Ghana have made significant investment and commitment towards vaccines procurement and logistics for vaccination. For example, the country has community health nurses who are involved in routine immunization programs, while there is significant and functional cold chain capacity including planned expansion for ultra-cold chain to accommodate COVID-19 vaccines such as Pfizer that require ultra-cold storage. Notwithstanding this, many LMICs fall short of the financial requirement for COVID-19 vaccination of their population. Therefore, donor partners, World Bank, and other regional development banks (e.g., African Development Bank) will have to introduce additional financing facilities that will support various countries’ vaccination plans. This could improve equity and access to COVID-19 vaccines and consolidate the gains of the global efforts to control the pandemic.

This study is limited mainly by the current prevailing global vaccine market conditions. There is generally unstable demand and supply of vaccines, which means that at any point in time a combination of vaccines based on availability may be used by the country, also affecting vaccine price. This will likely affect the cost estimates this study produced. In the foreseeable future, emerging issues relating to possible manufacturing of vaccines in Africa, among other considerations could have impacts on cost estimates. This is the first national level costing of COVID-19 vaccination programme conducted in Ghana using the CVIC tool, and one of the first in SSA. It, therefore, presents an opportunity to other countries to use this as basis and guide for their COVID-19 vaccination programme costing and budgeting. Other Ministries of Health, especially among the 92 Advance Market Commitment (AMC) eligible countries may be guided by this work to support their COVID-19 vaccination costing and budgeting. Again, it will be an important guide for financing and sustainability of the COVID-19 vaccination programme which can be integrated into the various countries’ NDVPs. Using the CVIC tool, this research could be replicated in other settings. It must be emphasized that data collection and populating the CVIC tool as evidenced in this work, is systematic and iterative; also requiring active engagement of relevant stakeholders including both policy makers and frontline actors.

The cost estimates are important for advocacy and mobilization of financial and other resources for COVID-19 vaccination. Therefore, it is important for government to use these estimates in fundraising discussions with development partners/donors – first, government needs to realistically identify what proportion of the costs or what categories of costs could be borne domestically, as a way to motivate development partner support with the remaining costs. Given that the CVIC tool was developed by international partners and supported by major global health organizations, government needs to make a conscious effort to be consistent in the cost estimates put forward for various discussions, and the results produced in this study are useful for that purpose. To date, varying estimates of costs have been mentioned (in the media, to development partners, etc.), some of which do not include all the categories required for a comprehensive analysis. The estimates generated from the CVIC and reported here are comprehensive and ensure that critical cost elements are not ignored in the quest to raise financial and other resources required for COVID-19 vaccination.

## 5 Conclusion

This study has estimated the cost of COVID-19 vaccine introduction and deployment in Ghana to be between $348.7 and $436.1 million for the target population of 17.5 million, translating into per person completed primary schedule cost of $20.9–$26.2 and per dose (including vaccine cost) of $10.5–$13.1. Again, per person completed primary schedule excluding vaccine cost was $4.5 and $4.6, thus per dose excluding vaccine also ranged from $2.2 to $2.3. The main cost driver was vaccine doses, including shipping, which accounts for between 78% and 83% of total cost. Further, an estimated 8,437–10,247 vaccinators (non-FTEs) would be required during this period to vaccinate using a mix of delivery strategies, accounting for 8–10% of total cost.

## Funding

Funding for the workshops and working sessions was provided by the Ministry of Health, Ghana. Stephen Resch was supported by the Bill and Melinda Gates Foundation.

## Author contribution

All authors made substantial contributions to the conception and design of the study, or acquisition of data, or analysis and interpretation of data, drafting the article or revising it critically for important intellectual content, and final approval of the manuscript.

## Declaration of Competing Interest

The authors declare the following financial interests/personal relationships which may be considered as potential competing interests: Justice Nonvignon reports administrative support was provided by Ghana Ministry of Health. Stephen C. Resch reports article publishing charges was provided by Bill & Melinda Gates Foundation.

## Footnotes

2 https://www.who.int/publications/i/item/who-2019-ncov-vaccine_deployment_tool-2021.1.

3 https://www.who.int/publications/i/item/WHO-2019-nCoV-Vaccine-introduction-RA-Tool-2020.1.

4 https://www.who.int/publications/i/item/WHO-2019-nCoV-Vaccine-deployment-2021.1-eng.

5 https://www.who.int/publications/i/item/who-2019-ncov-vaccine_deployment_tool-2021.1.

6 https://www.unicef.org/supply/covid-19-vaccine-market-dashboard.
